# Methodological Quality of Consensus Guidelines in Implant Dentistry

**DOI:** 10.1371/journal.pone.0170262

**Published:** 2017-01-20

**Authors:** Clovis Mariano Faggion, Karol Apaza, Tania Ariza-Fritas, Lilian Málaga, Nikolaos Nikitas Giannakopoulos, Marco Antonio Alarcón

**Affiliations:** 1 Department of Periodontology and Operative Dentistry, Faculty of Dentistry, University of Münster, Münster, Germany; 2 Academic Department of Clinical Stomatology, Section of Integral Oral Implantology, Cayetano Heredia Peruvian University, Lima, Perú; 3 Academic Department of Clinical Stomatology, Section of Periodontology and Implants, Cayetano Heredia Peruvian University, Lima, Perú; 4 Department of Prosthodontics, Faculty of Medicine, University of Würzburg, Würzburg, Germany; Virginia Commonwealth University, UNITED STATES

## Abstract

**Background:**

Consensus guidelines are useful to improve clinical decision making. Therefore, the methodological evaluation of these guidelines is of paramount importance. Low quality information may guide to inadequate or harmful clinical decisions.

**Objective:**

To evaluate the methodological quality of consensus guidelines published in implant dentistry using a validated methodological instrument.

**Methods:**

The six implant dentistry journals with impact factors were scrutinised for consensus guidelines related to implant dentistry. Two assessors independently selected consensus guidelines, and four assessors independently evaluated their methodological quality using the Appraisal of Guidelines for Research & Evaluation (AGREE) II instrument. Disagreements in the selection and evaluation of guidelines were resolved by consensus. First, the consensus guidelines were analysed alone. Then, systematic reviews conducted to support the guidelines were included in the analysis. Non-parametric statistics for dependent variables (Wilcoxon signed rank test) was used to compare both groups.

**Results:**

Of 258 initially retrieved articles, 27 consensus guidelines were selected. Median scores in four domains (applicability, rigour of development, stakeholder involvement, and editorial independence), expressed as percentages of maximum possible domain scores, were below 50% (median, 26%, 30.70%, 41.70%, and 41.70%, respectively). The consensus guidelines and consensus guidelines + systematic reviews data sets could be compared for 19 guidelines, and the results showed significant improvements in all domain scores (*p* < 0.05).

**Conclusions:**

Methodological improvement of consensus guidelines published in major implant dentistry journals is needed. The findings of the present study may help researchers to better develop consensus guidelines in implant dentistry, which will improve the quality and trust of information needed to make proper clinical decisions.

## Introduction

Consensus guidelines are important tools that help clinicians make appropriate decisions in the treatment of their patients. The developers of these guidelines suggest recommendations for clinical practice based on the best available evidence from, for example, well-conducted systematic reviews [1]. Consensus guidelines aim to promote better clinical treatment based on the weighing of potential benefits and harms, resources available, patients’ preferences, and scientific evidence [2]. To reach this goal, guidelines should be developed at the highest methodological level possible. Those based on low-quality, biased methodologies will likely guide clinicians to make ineffective and potentially harmful clinical decisions. Thus, evaluation of the methodological quality of consensus guidelines is important in any field.

Consensus guidelines are usually planned and developed by leading experts in the respective field who personally meet to discuss recommendations for clinical practice. As the name suggests, there is always an attempt for reaching consensus in generating clinical recommendations. This approach does not vary too much from the development of so-called “classic guidelines” which are also produced by specialists in the field of the respective guideline. For example, the Cochrane Collaboration defines a clinical guideline as “a systematically developed statement for practitioners and participants about appropriate health care for specific clinical circumstances” [http://community-archive.cochrane.org/glossary/]. Thus, although one can argue that might have slight methodological differences between a consensus and a “classic” guideline, the objective of both guidelines is exactly the same: the improvement of health care of our patients. Therefore, both documents require to be scrutinized for their quality.

The Appraisal of Guidelines Research and Evaluation (AGREE) tool is a validated instrument used to evaluate the methodological quality and transparency of development of clinical guidelines. This tool was first published in 2003 [3], and the most recent version (AGREE II) [4] has been refined and updated with better methodological properties [5]. Both versions of the instrument have been used in a variety of medical disciplines [6–8]. To our knowledge, however, the methodological quality of consensus guidelines in implant dentistry has not been evaluated.

The main objective of this study was to evaluate the methodological quality of consensus guidelines published in highly ranked implant dentistry journals using the AGREE II tool. A secondary objective was to evaluate whether the inclusion of systematic reviews conducted to support the consensus guidelines improved the methodological quality of the consensus guidelines.

## Material and Methods

### Study question

This methodological study was performed to answer the question, “Do consensus guidelines published in highly ranked implant dentistry journals meet the requirements proposed in the AGREE II instrument?”

### Eligibility criteria

Consensus guidelines on implant dentistry published since 2009 (with the respective consensus conference held after May 2009) in the six major implant dentistry journals (listed below) were included. Other types of document, such as those related to primary and secondary research, were excluded. Data from systematic reviews conducted to support the consensus guidelines were also included in the second part of the assessment.

### Search

Two authors (KA, MA) independently searched for consensus guidelines in the six implant dentistry journals with impact factors (2014) assigned by Journal Citation Reports (http://ipscience.thomsonreuters.com/product/journal-citation-reports/?utm_source=false&utm_medium=false&utm_campaign=false): *Clinical Oral Implants Research* (COIR), *Clinical Implant Dentistry and Related Research* (CIDRR), *European Journal of Oral Implants* (EJOI), *The International Journal of Oral and Maxillofacial Implants* (JOMI), *Journal of Oral Implantology*, and *Implant Dentistry*. Searches were limited to guidelines published between May 2009 and February 2016. The Medline database was also searched (via PubMed), using the following key words and Boolean operators: ‘guidelines’ OR ‘consensus’ OR ‘position paper’ OR ‘workshop’ OR ‘proceeding’ OR ‘conference’ in combination (AND) with each of the six journal titles. This second search was conducted to provide a detailed pathway for reporting of the literature search process.

### Selection of reports

First, two authors (KA, MA) evaluated the titles and abstracts of reports to determine eligibility for initial inclusion. Then, they scrutinised full texts of papers to determine whether the studies met the inclusion criteria. The authors documented excluded articles, with corresponding reasons for exclusion. The two authors performed study selection independently and in duplicate, and discussed any disagreement regarding the inclusion or exclusion of papers until consensus was achieved.

### The AGREE II instrument

The AGREE II tool is an updated version of the seminal AGREE tool developed by the AGREE Collaboration [3], a group of researchers and guideline developers. It consists of 23 items in six domains (Table 1), used mainly to evaluate the methodological rigour and transparency of guidelines [5]. Items are rated using a seven-point scale ranging from ‘strongly disagree’ to ‘strongly agree’, representing the assessor’s confidence in whether the guidelines meet the quality of reporting and AGREE criteria. Each domain score is calculated by summing component item scores and scaling the value as a percentage of the maximum possible score, according to the developer’s instructions. As the AGREE II tool was made publicly available in May 2009, only consensus guidelines published from this year forward (with the respective consensus conference held after May 2009) were included in the present study.

**Table 1:** AGREE II items.

DOMAINS	ITEM LIST
DOMAIN 1: SCOPE AND PURPOSE	1. The overall objective(s) of the guideline is (are) specifically described.
	2. The health question(s) covered by the guideline is (are) specifically described.
	3. The population (patients, public, etc.) to whom the guideline is meant to apply is specifically described.
DOMAIN 2: STAKEHOLDER INVOLVEMENT	4. The guideline development group includes individuals from all relevant professional groups.
	5. The views and preferences of the target population (patients, public, etc.) have been sought.
	6. The target users of the guideline are clearly defined
DOMAIN 3: RIGOUR OF DEVELOPMENT	7. Systematic methods were used to search for evidence.
	8. The criteria for selecting the evidence are clearly described
	9. The strengths and limitations of the body of evidence are clearly described
	10. The methods for formulating the recommendations are clearly described.
	11. The health benefits, side effects, and risks have been considered in formulating the recommendations.
	12. There is an explicit link between the recommendations and the supporting evidence.
	13. The guideline has been externally reviewed by experts prior to its publication.
	14. A procedure for updating the guideline is provided.
DOMAIN 4: CLARITY OF PRESENTATION	15. The recommendations are specific and unambiguous.
	16. The different options for management of the condition or health issue are clearly presented
	17. Key recommendations are easily identifiable
DOMAIN 5: APPLICABILITY	18. The guideline describes facilitators and barriers to its application.
	19. The guideline provides advice and/or tools on how the recommendations can be put into practice.
	20. The potential resource implications of applying the recommendations have been considered.
	21. The guideline presents monitoring and/or auditing criteria.
DOMAIN 6: EDITORIAL INDEPENDENCE	22. The views of the funding body have not influenced the content of the guideline.
	23. Competing interests of guideline development group members have been recorded and addressed.

### Data evaluation

Four authors (KA, TA, LM, and MA) independently applied the AGREE II tool, first to consensus guidelines only, and then with the inclusion of systematic reviews conducted to support the guidelines. The latter assessment was performed to understand the amount of information added to clinical recommendations by the consideration of systematic reviews as supporting material. Disagreements on data evaluation were resolved by discussion among the four authors until consensus was achieved.

### Assessor training

A standardised form containing the 23 AGREE II items was produced for data extraction/evaluation. After carefully reading the AGREE handbook, the four assessors applied the tool to evaluate the methodology of consensus guidelines not included in the present study, recording data in the form. Between rounds of data evaluation, assessors discussed the outcomes comprehensively to improve the homogeneity of assessment.

### Data analysis

Domains scores were presented as medians of percentages of maximum possible scores with their respective interquartile range (IQR). Domain scores from the two data sets (consensus guidelines and consensus guidelines plus supporting systematic reviews) were compared using non-parametric statistics for dependent variables (Wilcoxon signed rank test), with the level of significance set at *p* = 0.05. Statistical analyses were performed with the SigmaPlot software (version 12.0 for Windows; Systat Software GmbH, Erkrath, Germany).

## Results

### Number of consensus guidelimatic reviews supporting the guidelines. t/disagreement ratings between consensus guidelines and consensus guidnes

**Fig 1:**
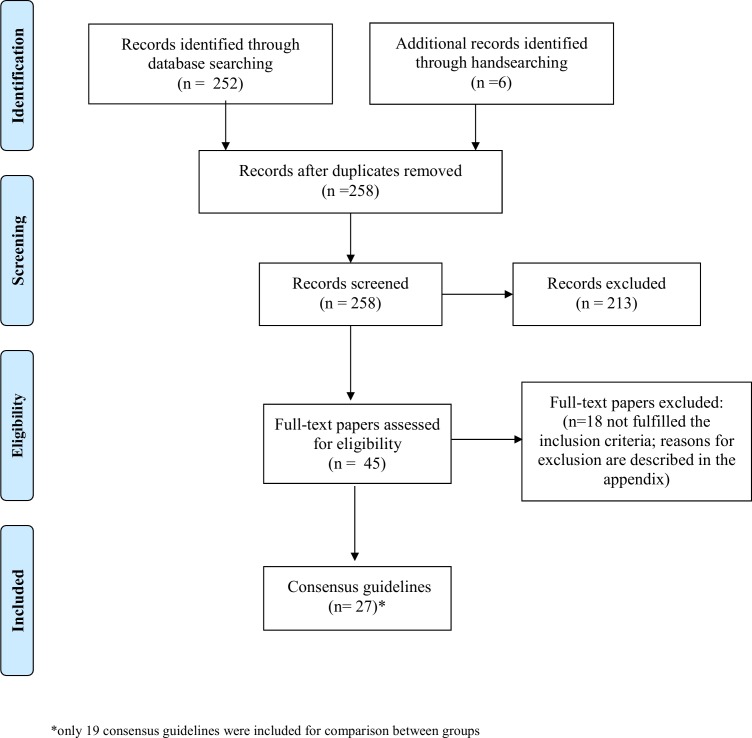
PRISMA-style flow diagram of the consensus guidelines search process.

We initially identified 258 publications. After the assessment of titles and abstracts, 213 publications were excluded. Full text evaluation led to the exclusion of 45 additional publications. Hence, 27 consensus guidelines were included. The literature search process is illustrated in Fig 1, and publications included in and excluded from the analysis are listed in the supplementary information in S1 and S2 Appendix, respectively.

### Characteristics of consensus guidelines

Consensus guidelines were published in five of the six journals searched: COIR (*n* = 12), JOMI (*n* = 7), EJOI (*n* = 6), CIDRR (*n* = 1), and *Implant Dentistry* (*n* = 1). Twenty-six guidelines were developed after meetings held in European countries. The number of authors of the consensus guidelines ranged from 2 to 27 (median, 9). The European Association for Osseointegration was the organisation that most frequently supported the meetings and development of consensus guidelines (*n* = 9). Table 2 provides detailed information on the characteristics of consensus guidelines included in this study.

**Table 2:** Characteristics of the consensus guidelines included in this study.

Consensus Guideline	Year of publication	Name of the meeting	Place of the meeting	Topic of the consensus guideline	Journal	Number of authors	Sponsor	With systematic review?
Schwarz et al.	2016	Camlog Foundation Consensus Report 2015	Barcelona, Valencia, Spain	Loading protocols and implant supported restorations proposed for the rehabilitation of partially and fully edentulous jaws	COIR	7	Camlog Foundation	Yes
Sanz et al.	2015	The 4th EAO Consensus Conference 2015	Schwyz, Switzerland	Therapeutic concepts and methods for improving dental implant outcomes	COIR	12	EAO	Yes
Sicilia et al.	2015	The 4th EAO Consensus Conference 2015	Schwyz, Switzerland	Long-term stability of peri-implant tissues after bone or soft tissue augmentation. Effect of zirconia or titanium abutments on peri-implant soft tissues	COIR	15	EAO	Yes
Hämmerle et al.	2015	The 4th EAO Consensus Conference 2015	Schwyz, Switzerland	Digital technologies to support planning, treatment, and fabrication processes and outcome assessments in implant dentistry. Summary and consensus statements. The 4th EAO consensus conference 2015	COIR	17	EAO	Yes
Klinge et al.	2015	The 4th EAO Consensus Conference 2015	Schwyz, Switzerland	The patient undergoing implant therapy	COIR	15	EAO	Yes
Schwarz et al.	2014	Camlog Foundation Consensus Report 2013	Rome, Italy	Impact of implant-abutment connection, positioning of the machined collar/microgap, and platform switching on crestal bone level changes	COIR	7	Camlog Foundation	Yes
A Foundation for Oral Rehabilitation (FOR) consensus conference	2014	Foundation for Oral Rehabilitation (FOR) consensus conference 2014	University of Mainz, Germany	Patient-centred rehabilitation of edentulism with an optimal number of implants	EJOI	11	Foundation for Oral Rehabilitation	Yes
Heitz-Mayfield et al.	2014	Fifth ITI Consensus conference 2013-Group 5	Bern, Switzerland	Prevention and management of biologic and technical implant complications	JOMI	23	ITI	Yes
Gallucci et al.	2014	Fifth ITI Consensus conference 2013-Group 4	Bern, Switzerland	Implant loading protocols	JOMI	23	ITI	Yes
Morton et al.	2014	Fifth ITI Consensus conference 2013-Group 3	Bern, Switzerland	Optimizing esthetic outcomes in implant dentistry	JOMI	21	ITI	Yes
Wismeijer et al.	2014	Fifth ITI Consensus conference 2013-Group 2	Bern, Switzerland	Restorative materials and techniques for implant dentistry	JOMI	21	ITI	Yes
Bornstein et al.	2014	Fifth ITI Consensus conference 2013-Group 1	Bern, Switzerland	Contemporary surgical and radiographic techniques in implant dentistry	JOMI	27	ITI	Yes
Albrektsson et al.	2012	Estepona consensus meeting, 2012	Malaga, Spain	Peri-implantitis	CIDRR	12	Not reported	No
Harris et al.	2012	A consensus workshop organized by the European Association for Osseointegration at the Medical University of Warsaw	Warsaw, Poland	Diagnostic imaging in implant dentistry	COIR	9	EAO	No
Gotfredsen et al.	2012	The Third EAO Consensus Conference	Schwyz, Switzerland	Reconstructions on implants	COIR	16	EAO	Yes
Sicilia et al.	2012	The Third EAO Consensus Conference	Schwyz, Switzerland	Computer-guided implant therapy. Soft and tissue aspects	COIR	15	EAO	Yes
Klinge et al.	2012	The Third EAO Consensus Conference	Schwyz, Switzerland	Peri-implant tissue destruction.	COIR	14	EAO	Yes
Albrektsson et al.	2012	The Third EAO Consensus Conference	Schwyz, Switzerland	Implant survival and complications.	COIR	15	EAO	Yes
Benavides et al.	2012	International Congress of Oral Implantologists consensus report.	Seoul, Korea,	Use of cone beam computed tomography in implant dentistry	Implant dentistry	15	ICOI	No
Hämmerle et al.	2012	Osteology Consensus Group 2011	Vienna, Austria	The biology and treatment of extraction sockets.	COIR	3	OCG	Yes
Esposito et al.	2012	Stockholm Consensus	Stockholm, Sweden	Treatment Options for the Maintenance of Marginal Bone Around	EJOI	9	Not reported	Yes
Academy of Osseointegration	2010	Guidelines of the Academy of Osseointegration	Not reported	The provision of dental implants and associated patient care.	JOMI	Not reported	AO	No
Klein et al.	2011	1st DGI Consensus Conference	Aerzen, Germany	Use of bone substitute materials	EJOI	9	DGI	Yes
Schley et al.	2011	1st DGI Consensus Conference	Aerzen, Germany	Prosthetic treatment	EJOI	9	DGI	Yes
Nitsche et al.	2011	1st DGI Consensus Conference	Aerzen, Germany	X-Ray diagnostic and image based computerized	EJOI	10	DGI	Yes
Weng et al.	2010	1st DGI Consensus Conference	Aerzen, Germany	Ridge preservation	EJOI	10	DGI	Yes
Albrektsson et al.	2013	Estepona consensus meeting, 2012	Estepona, Spain	Bone loss around dental implants	CIDRR	3	Straumann, Astra Tech, Dentsply, Nobel Biocare, Biomet3i.	No

COIR: Clinical Oral Implants Research; EJOI: European Journal of Oral Implantology; JOMI: International Journal of Oral and Maxillofacial Implants; CIDDR: Clinical Implant Dentistry and Related Research; ICOI: International Congress of Oral Implantologists; OCG: Osteology Consensus Group; AO: Academy of Osseointegration; DGI: German Association of Implantology.

### Methodological quality of guidelines

#### Consensus guidelines only

The score for domain 4 (clarity of presentation) was highest (median, 75; IQR 15.30), followed by the score for domain 1 (scope and purpose: median, 69.40; IQR, 36.20). Median scores for domains 2 (stakeholder involvement) and 6 (editorial independence) were both 41.70 (IQRs, 17.70 and 83.30, respectively). Scores for domains 3 (rigour of development) and 5 (applicability) were lowest (median, 30.70 [IQR, 26.50] and 26 [IQR, 12.50], respectively).

#### Consensus guidelines plus systematic reviews

When systematic reviews were included in the sample, the score for domain 1 was highest (median, 84.70; IQR, 9.80). The median score for domain 6 was second highest (79.20; IQR, 73), but this score showed the greatest variability among guidelines. The third highest score was for domain 4 (median, 76.40; IQR, 18.10), followed by the scores for domains 3 and 2 (median, 56.30 [IQR, 34.40] and 50 [IQR, 44.40], respectively). The score for domain 5 was lowest (median, 26; IQR, 20.80). Tables in S2 and S3 Tables report the complete AGREE II scores for consensus guidelines alone and consensus guidelines + systematic reviews, respectively. Table in S3 Table shows domain scores for the 19 consensus guidelines with no difference in hierarchy between data sets.

#### Group comparisons

Domain scores could be compared between data sets for 19 of the 27 consensus guidelines (Table in S4 Table). Scores differed significantly for all domains (*p* < 0.05; Table 3).

**Table 3:** Comparison of AGREE II scores (percentage of the maximum possible score for a specific domain) between consensus guidelines alone and consensus guidelines + systematic reviews. CG: consensus guideline; CGSR: consensus guideline + systematic review). Scores are related to 19 possible comparisons. The p values refer to Wilcoxon Signed Rank Test for paired samples.

Domain 1		Domain 2		Domain 3		Domain 4		Domain 5		Domain 6	
CG	CGSR	CG	CGSR	CG	CGSR	CG	CGSR	CG	CGSR	CG	CGSR
40.28	62.50	41.67	50	39.59	57.81	55.56	66.7	26.04	34.38	31.25	66.67
80.56	86.11	34.72	84.72	65.62	86.98	75	77.78	15.63	57.29	81.25	100
80.56	88.89	33.33	50	43.75	83.33	83.33	90.28	36.46	61.46	41.67	45.83
50	86.11	40.28	86.11	67.71	81.25	83.33	88.89	41.67	62.5	41.67	87.5
36,11	81.94	44.44	70.83	73.96	85.42	86.11	100	35.42	62.5	41.67	85.42
44.44	55.56	33.33	48.61	40.63	60.94	50	68.06	26.04	37.5	27.08	66.67
6.94	87.5	41.67	56.94	19.27	50.52	34.72	47.22	2.08	6,25	2.08	20.83
73.61	84.72	50	48.61	18.23	47.40	70.83	76.39	31.25	31.25	95.83	95.83
75	83.33	50	45.83	19.79	47.40	73.61	75	25	26.04	95.83	93,75
72.22	84.72	47.22	40.28	18.23	47.92	75	73.61	25	26.04	95.83	91.67
83.33	81.94	44.44	41.67	18.23	46.88	70.83	77.78	26.04	25	97.92	97.92
83.33	83.33	43.06	40.28	19.79	50	72.22	75	27.08	27.08	97.92	97.93
11.11	29.17	16.67	50	7.81	29.69	38.89	76.39	16.67	29.17	6.25	4.17
75	81.94	27.78	26.39	45.83	46.35	80.56	87.5	23.96	2604	0	18.75
69.44	80.56	27.78	38.89	27.08	36.46	61.11	68.06	17.71	16.67	27.08	18.75
69.44	73.61	25	30.56	27.60	38.54	76.39	83.33	29.17	25	4.17	10.42
72.22	83.33	22.22	34.72	34.38	37.5	80.56	79.17	26.04	25	4.17	1042
88.89	91.67	41.67	47.22	71.35	79.69	81.94	90.28	38.54	43.75	89.59	100
87.5	91.67	83.33	91.67	67.19	76.04	87.5	82	22.92	37.5	68.75	77.08
P<0.001		P = 0.002		P<0.001		P<0.001		P = 0.003		P = 0.003	

## Discussion

### Brief summary of findings

In this sample of 27 consensus guidelines in implant dentistry, median scores for four AGREE II domains (stakeholder involvement, rigour of development, applicability, and editorial independence) were less than 50. However, the inclusion of supporting systematic reviews significantly improved all domain scores. Great variability was found among consensus guidelines, as reflected by large IQRs for some domains.

### Implications of the present findings

The present findings have important consequences for the further development of consensus guidelines in implant dentistry. First, they provide a measurement of the methodological quality of these guidelines that may greatly impact clinicians’ decisions. Consensus guidelines in the present sample were supported by reputable implant dentistry organisations, and were published in highly ranked implant dentistry journals, which are reliable sources of information for clinicians working with dental implants. Second, the findings provide comprehensive information about which domains should be prioritised in the development of future guidelines in this field. Third, this study demonstrated that the AGREE II tool can serve as a reference for the development of future consensus guidelines in implant dentistry.

In the present study, scores for domain 5 (applicability) were lowest. These results show that a gap currently exists between the evidence provided and its applicability in the clinical setting. Scores for domain 3 (rigour of development), which more directly reflects the methodological aspects of the guidelines, were second lowest. Importantly, scores for domain 2 (stakeholder involvement) were also low. For example, the sub-item ‘the views and preferences of the target population (patients, public, etc.) have been sought’ was poorly addressed in all consensus guidelines. Patients’ views are pivotal in gaining an understanding of their needs, and future guidelines in implant dentistry should include more information from patients’ perspectives. One approach would be to select patients to attend or participate in consensus meetings.

The significant improvement in all domain scores achieved by the inclusion of systematic reviews suggests that these reviews contain much important information needed to evaluate the methodological quality of consensus guidelines. We thus recommend that users examine both types of material to more fully understand the quality of guidelines. Ideally, systematic reviews and guidelines are produced at the highest methodological level possible and published separately, with the reviews serving as the source for guideline development [1].

### Comparison with other studies

Few publications describe the use of the AGREE II tool to evaluate clinical guidelines in dentistry. Horner et al. [9] recently evaluated 26 guidelines on the use of cone-beam computerised tomography in dental and maxillofacial radiology using the AGREE II instrument. As in the present analysis, they obtained good scores for domain 1 (scope and purpose) and very poor scores for domain 5 (applicability) [9]. San Martin-Galindo et al. [10] used the AGREE II tool to evaluate three guidelines on the use of pit and fissure sealants for dental clinicians; scores for domain 6 (editorial independence) were lowest. In the present study, this domain score was the third lowest when the consensus guidelines were evaluated alone. These findings may reflect the lack of good reporting of potential conflicts of interest by parties involved in guideline development. A few other studies have evaluated guidelines in dentistry using the original AGREE instrument [6,11–14]. Most of these studies showed that guidelines were of low quality.

### Strengths and limitations of the present study

To our knowledge, this study is the first to evaluate the methodological quality of consensus guidelines published in highly ranked implant dentistry journals using a validated tool. The AGREE II instrument represents improvement over the original AGREE tool, enabling more in-depth evaluation of the strengths and weaknesses of guidelines, and it has shown validity and reliability [15,16]. Thus, this evaluation of the quality of consensus guidelines was probably conducted with the best methodological tool available.

One may argue that AGREE II instrument is an inadequate methodology for assessing consensus guidelines. The idea is that consensus guidelines are developed by experts attending workshops and they do not fulfil the requirements of a quality instrument. But, this is the main reason for applying an instrument such as AGREE II. The question here is: what is the validity of a document that does not allow audit and quality evaluation? Therefore, we understand this approach is appropriate for several reasons. Firstly, guidelines included in the study fall within the Cochrane Collaboration’s definition of a clinical guideline as ‘a systematically developed statement for practitioners and participants about appropriate health care for specific clinical circumstances’ [17]. Secondly, the AGREE II handbook reports that the instrument is “generic” and can be applied to a great variety of documents. Thirdly, the literature contains several reports on the use of the AGREE instrument to evaluate consensus guidelines in other medical fields [8, 18–24]. Fourthly, and finally, the consensus guidelines included in this sample were developed by key-people in the respective field, and dental practitioners will likely follow them to make clinical decisions. So, the effect is the same of a considered “standard” guideline. In the end, clinicians will use the document for improving clinical treatments. Hence, the focus should be on whether the document includes recommendations for clinical action, instead on its structure or how it was developed.

We statistically compared evaluations performed with and without additional information from systematic reviews supporting the consensus guidelines. However, some limitations should be kept in mind when interpreting these results. Firstly, adequate sample size was difficult to determine, and our sample of guidelines is arguably small. Nevertheless, AGREE II domain scores showed robust and significant improvement with the inclusion of data from systematic reviews, and similar results would likely be obtained with a larger sample. Secondly, comparison is ideally performed between two independent groups. However, the identification of two sets of similar guidelines (in terms of structure and objectives) for a comparison like that performed in this study would be challenging. Although limited, this comparison is relevant because it provides quantitative evidence for the amount of information that supporting systematic reviews can add to consensus guidelines. In other words, the reader can understand these two different scenarios (guidelines with and without systematic review). More than focusing on *p* values, which might generate misleading assumptions [25], readers should observe the magnitude of changes in AGREE II scores.

In the present study, we did not attempt to determine which consensus guidelines are recommended for clinical practice and which are not. As reported in the AGREE II user’s manual (AGREE II instrument), the AGREE Collaboration does not recommend the application of any score threshold to differentiate between high-quality and poor-quality guidelines. They recommend that decisions about the use of guidelines be made by users, oriented by the context in which the AGREE II instrument is applied.

### Future development of consensus guidelines in implant dentistry

The consensus guidelines included in the present study were produced by key opinion leaders in the field of implant dentistry. We understand that the involvement of authorities, researchers, and clinicians in the development of such guidelines is important, as it represents integration between research foundation and clinical relevance. However, guidelines should be produced to the highest methodological quality possible, to give users more accurate information about the level and quality of evidence they intend to apply in the clinical setting.

Implant dentistry has reached a level of excellence in the conducting of systematic reviews. Now, it is time to move forward to improve the quality of clinical guidelines, which can provide a bridge between evidence and its applicability. The concept of developing consensus guidelines without a robust methodology is a remnant from the “pre–evidence-based” era. Hence, this methodological gap between well-developed systematic reviews and clinical practice guidelines should be reduced.

The so-called “classic” guidelines should also be scrutinized for quality with the AGREE II instrument. In the implant dentistry field they may also be available. For example, we searched Medline (via PubMed) for such guidelines (search strategy: dent*[ti] AND implant*[ti] AND guideline*[ti] AND 2009: 2016[dp], in 25th December 2016), and found 14 potential “non-consensus” implant dentistry guidelines. Although it is not in the scope of the present study to evaluate these guidelines, it would be also important to evaluate them in a future project.

## Conclusions

There is room to improve the quality of consensus guidelines published in highly ranked implant dentistry journals. Clinicians’ and researchers’ development of consensus guidelines to improve clinical treatment with dental implants is laudable. However, as for primary and secondary research, these guidelines should adhere to high and transparent standards. The AGREE II instrument can be used as a reference for the development of high-quality guidelines to provide unbiased and adequate clinical recommendations to clinicians working with dental implants.

## Supporting Information

S1 PRISMA Checklist (DOC)

S1 Appendix List of included consensus guidelines. (DOCX)

S2 Appendix List of excluded documents with reasons for exclusion. (DOCX)

S1 Table Evaluation of consensus guidelines published in high-ranked implant dentistry journals with the AGREE II instrument. (DOCX)

S2 Table Evaluation of consensus guidelines + systematic review published in high-ranked implant dentistry journals with the AGREE II instrument. (DOCX)

S3 Table Medians of percentages of the maximum possible score for the respective domains across consensus guidelines in implant dentistry (19 possible comparisons). IQR: interquartile range. CG: consensus guideline; CGSR: consensus guideline + systematic review.
(DOCX)

S4 Table Percentage of the maximum possible score for the respective domains across consensus guidelines in implant dentistry (DOCX)
