# Correction: Methodological Quality of Consensus Guidelines in Implant Dentistry

**DOI:** 10.1371/journal.pone.0173437

**Published:** 2017-03-02

**Authors:** Clovis Mariano Faggion, Karol Apaza, Tania Ariza-Fritas, Lilian Málaga, Nikolaos Nikitas Giannakopoulos, Marco Antonio Alarcón

The heading of the first paragraph of the Results section is incorrect. The correct heading should be Number of consensus guidelines.
