# Supplementary material for: Methodological Quality of Consensus Guidelines in Implant Dentistry
Source: PLoS One. 2017 Jan 20;12(1):e0170262. doi: 10.1371/journal.pone.0170262 (PMC5249121; doi:10.1371/journal.pone.0170262)
Supplement: S1 Appendix — (DOCX) [file pone.0170262.s002.docx]

**S1 Appendix**

1. Academy of Osseointegration. 2010. Guidelines of the Academy of Osseointegration for the provision of dental implants and associated patient care. The International Journal Oral Maxillofacial Implants 25(3): 620-627.

2. Albrektsson, T., Buser, D. & Sennerby, L. (2012) Crestal bone loss and oral implants. Clinical Implant Dentistry and Related Research.14(6): 783-91.

3. Albrektsson, T. & Donos, N. (2012) Working Group 1. Implant survival and complications. The Third EAO consensus conference 2012. Clinical Oral Implants Research 23(6): 63-65.

4. Albrektsson, T., Buser, D., Chen, ST., Cochran, D., DeBruyn, H., Jemt, T., Koka, S., Nevins, M., Sennerby, L., Simion, M., Taylor, TD. & Wennerberg, A. (2012) Statements from the Estepona consensus meeting on peri-implantitis, February 2-4, 2012. Clinical Implant Dentistry and Related Research 14(6): 781-782.

5. Benavides, E., Rios, HF., Ganz, SD., An CH, Resnik, R., Reardon, GT., Feldman SJ., Mah, JK., Hatcher, D., Kim, MJ., Sohn, DS., Palti, A., Perel, ML., Judy, KW., Misch, CE. & Wang, HL. (2012) Use of cone beam computed tomography in implant dentistry: the International Congress of Oral Implantologists consensus report. Implant Dentistry 21(2): 78-86.

6. Bornstein, MM., Al-Nawas, B., Kuchler, U. & Tahmaseb, A. (2014) Consensus statements and recommended clinical procedures regarding contemporary surgical and radiographic techniques in implant dentistry. The International Journal Oral Maxillofacial Implants 29: 78-82.

7. Esposito M, Klinge B, Meyle J, Mombelli A, Rompen E, van Steenberghe D, Van Dyke T, Wang HL & van Winkelhoff AJ. (2012) Working Group on the Treatment Options for the Maintenance of Marginal Bone Around Endosseous Oral Implants, Stockholm, Sweden, 8 and 9 September 2011. Consensus statements. European Journal of Oral Implantology 5:105-106.

8. Gallucci, GO., Benic, GI., Eckert, SE., Papaspyridakos, P., Schimmel, M., Schrott, A. & Weber, HP. (2014) Consensus statements and clinical recommendations for implant loading protocols. The International Journal Oral Maxillofacial Implants 29: 287-290.

9. Gotfredsen, K. & Wiskott, A. (2012) Working Group 4. Consensus report – reconstructions on implants. The Third EAO Consensus Conference 2012. Clinical Oral Implants Research. 23(6): 238-241.

10. Hämmerle, CH., Araújo, MG., Simion, M. (2012) Osteology Consensus Group 2011. Evidence-based knowledge on the biology and treatment of extraction sockets. Clinical Oral Implants Research 23(5): 80-82.

11. Hämmerle, CH., Cordaro, L., van Assche, N., Benic, GI., Bornstein, M., Gamper, F., Gotfredsen, K., Harris, D., Hürzeler, M., Jacobs, R., Kapos, T., Kohal, RJ., Patzelt, SB., Sailer, I., Tahmaseb, A., Vercruyssen, M. & Wismeijer, D. (2015) Digital technologies to support planning, treatment, and fabrication processes and outcome assessments in implant dentistry. Summary and consensus statements. The 4th EAO consensus conference. Clinical Oral Implants Research 26(11): 97-101.

12. Harris, D., Horner, K., Gröndahl, K., Jacobs, R., Helmrot, E., Benic, GI., Bornstein, MM., Dawood, A. & Quirynen, M. (2012) E.A.O. guidelines for the use of diagnostic imaging in implant dentistry 2011. A consensus workshop organized by the European Association for Osseointegration at the Medical University of Warsaw. Clinical Oral Implants Research 23(11): 1243-1253.

13. Heitz-Mayfield, LJ., Needleman, I., Salvi, GE. & Pjetursson, BE. (2014) Consensus statements and clinical recommendations for prevention and management of biologic and technical implant complications. The International Journal Oral Maxillofacial Implants 29: 346-350.

14. Klein, MO. & Al-Nawas, B. (2011) For which clinical indications in dental implantology is the use of bone substitute materials scientifically substantiated? Systematic review, consensus statements and recommendations of the 1st DGI Consensus Conference in September 2010, Aerzen, Germany. European Journal of Oral Implantology 4: 11-29.

15. Klinge, B., Flemming, T., Cosyn, J., De Bruyn, H., Eisner, BM., Hultin, M., Isidor, F., Lang, NP., Lund, B., Meyle, J., Mombelli, A., Navarro, JM., Pjetursson, B., Renvert, S. & Schliephake, H. (2015) The patient undergoing implant therapy. Summary and consensus statements. The 4th EAO Consensus Conference 2015. Clinical Oral Implants Research 26(11): 64-67

16. Klinge, B. & Meyle, J. (2012) Working Group 2. Peri-implant tissue destruction. The Third EAO Consensus Conference. Clinical Oral Implants Research 23(6): 108-110.

17. Morton, D., Chen, ST., Martin, WC., Levine, RA. & Buser, D. (2014) Consensus statements and recommended clinical procedures regarding optimizing esthetic outcomes in implant dentistry. The International Journal Oral Maxillofacial Implants 29: 216-220.

18. Nitsche, T., Menzebach, M. & Wiltfang, J. (2011) What are the indications for three-dimensional X-ray-diagnostics and image-based computerised navigation aids in dental implantology? Systematic review, consensus statements and recommendations of the 1st DGI Consensus Conference in September, Aerzen, Germany. European Journal of Oral Implantology 4: 49-58.

19. Patient-centred rehabilitation of edentulism with an optimal number of implants. A Foundation for Oral Rehabilitation (FOR) consensus conference. European Journal of Oral Implantology. 2014; 7(2): 235-238.

20. Sanz, M., Donos, N., Alcoforado, G., Balmer, M., Gurzawska, K., Mardas, N., Milinkovic, I., Nisand, D., Rocchietta, I., Stavropoulos, A., Thoma, DS., & Torsello, F. (2015) Therapeutic concepts and methods for improving dental implant outcomes. Summary and consensus statements. The 4th EAO Consensus Conference 2015. Clinical Oral Implants Research 26(11): 202-206.

21. Schwarz, F., Alcoforado, G., Nelson, K., Schaer, A., Taylor, T., Beuer, F. & Strietzel, FP. (2014) Impact of implant-abutment connection, positioning of the machined collar/microgap, and platform switching on crestal bone level changes. Camlog Foundation Consensus Report. Clinical Implant Dentistry and Related Research 25(11): 1301-1303.

22. Schwarz, F., Sanz-Martín, I., Kern, JS., Taylor, T., Schaer, A., Wolfart, S. & Sanz, M. (2016) Loading protocols and implant supported restorations proposed for the rehabilitation of partially and fully edentulous jaws. Camlog Foundation Consensus Report. Clinical Oral Implants Research 27(8): 988-92.

23. Sicilia, A. & Botticelli, D. (2012) Working Group 3. Computer-guided implant therapy and soft- and hard-tissue aspects. The Third EAO Consensus Conference 2012. Clinical Oral Implants Research 23(6): 157-161.

24. Sicilia, A., Quirynen, M., Fontolliet, A., Francisco, H., Friedman, A., Linkevicius, T., Lutz, R., Meijer, HJ., Rompen, E., Rotundo, R., Schwarz, F., Simion, M., Teughels, W., Wennerberg, A. & Zuhr, O. (2015) Long-term stability of peri-implant tissues after bone or soft tissue augmentation. Effect of zirconia or titanium abutments on peri-implant soft tissues. Summary and consensus statements. The 4th EAO Consensus Conference 2015. Clinical Oral Implants Research 26(11): 148-152.

25. Schley, JS. & Wolfart, S. (2011) Which prosthetic treatment concepts present a reliable evidence-based option for the edentulous maxilla related to number and position of dental implants? Systematic review, consensus statements and recommendations of the 1st DGI Consensus Conference in September 2010, Aerzen, Germany. European Journal of Implantology. 4: 31-47.

26. Weng, D., Stock, V. & Schliephake, H. (2010) Are socket and ridge preservation techniques at the day of tooth extraction efficient in maintaining the tissues of the alveolar ridge? Systematic review, consensus statements and recommendations of the 1st DGI Consensus Conference in September Aerzen Germany. European Journal of Oral Implantology 4: 59-66.

27. Wismeijer D., Brägger U., Evans C., Kapos T., Kelly JR., Millen C., Wittneben JG., Zembic A. & Taylor TD. (2014) Consensus statements and recommended clinical procedures regarding restorative materials and techniques for implant dentistry. The International Journal Oral Maxillofacial Implants 29: 137-140.
