# Supplementary material for: Methodological Quality of Consensus Guidelines in Implant Dentistry
Source: PLoS One. 2017 Jan 20;12(1):e0170262. doi: 10.1371/journal.pone.0170262 (PMC5249121; doi:10.1371/journal.pone.0170262)
Supplement: S2 Appendix — (DOCX) [file pone.0170262.s003.docx]

**S2 Appendix**

1. Heitz-Mayfield LJ, Needleman I, Salvi GE, Pjetursson BE. Consensus Statements and Clinical Recommendations for Prevention and Management of Biologic and Technical Implant Complications. Int J Oral Maxillofac Implants. 2013 Aug 15.

Reason: Duplicate from Heitz-Mayfield et al. 2014

2. Gallucci GO, Benic GI, Eckert SE, Papaspyridakos P, Schimmel M, Schrott A, Weber HP. Consensus Statements and Clinical Recommendations for Implant Loading Protocols. Int J Oral Maxillofac Implants. 2013 Aug 15.

Reason: Duplicate from Gallucci et al 2014

3. Morton D, Chen ST, Martin WC, Levine R, Buser D. Consensus Statements and Recommended Clinical Procedures Regarding Optimizing Esthetic Outcomes in Implant Dentistry. Int J Oral Maxillofac Implants. 2013 Aug 15.

Reason: Duplicate from Morton et al 2014

4. Wismeijer D, Bragger U, Evans C, Kapos T, Kelly R, Millen C, Wittneben J, Zembic A, Taylor TD. Consensus Statements and Recommended Clinical Procedures Regarding Restorative Materials and Techniques for Implant Dentistry. Int J Oral Maxillofac Implants. 2013 Aug 15.

Reason: Duplicate from Wismeijer et al 2014

5. Proceedings of the 2nd EAO (European Association for Osseointegration) Consensus Conference, 19-22 February 2009, Pfäffikon, Switzerland. Clin Oral Implants Res. 2009;20(4):1-231.

Reason: Consensus Conference in 19-22 February 2009 (before AGREE II was publicly available)

6. Lang NP, Jepsen S; Working Group 4. Implant surfaces and design (Working Group 4). Clin Oral Implants Res. 2009 Sep;20(4):228-231.

Reason: Consensus Conference in 19-22 February 2009 (before AGREE II was publicly available)

7. Klinge B, Flemmig TF; Working Group 3. Tissue augmentation and esthetics(Working Group 3). Clin Oral Implants Res.2009;20(4):166-170.

Reason: Consensus Conference in 19-22 February 2009 (before AGREE II was publicly available)

8. Sanz M, Naert I; Working Group 2. Biomechanics/risk management (Working Group 2). Clin Oral Implants Res. 2009;20(4):107-111.

Reason: Consensus Conference in 19-22 February 2009 (before AGREE II was publicly available)

9. Hobkirk JA, Wiskott HW; Working Group 1. Ceramics in implant dentistry (Working Group 1). Clin Oral Implants Res. 2009;20 Suppl 4:55-57.

Reason: Consensus Conference in 19-22 February 2009 (before AGREE II was publicly available)

10. Hämmerle CH, Quirynen M. The Second EAO Consensus Conference 19-22 February 2009, Pfäffikon, Switzerland. Preface. Clin Oral Implants Res. 2009 Sep;20(4):1.

Reason: Consensus Conference in 19-22 February 2009 (before AGREE II was publicly available)

11. Proceedings of the 3rd EAO Consensus Conference, 15–18 February 2012, Pfäffikon, Schwyz, Switzerland. Clin Oral Implants Res. 2012 Oct;23(6):1-241.

Reason: Introductory part of the consensus

12. Klinge B, van Steenberghe D. Working Group on Treatment Options for the Maintenance of Marginal Bone Around Endosseous Oral Implants, Stockholm, Sweden, 8 and 9 September 2011. Methodology. Eur J Oral Implantol. 2012;5:9-12.

Reason: Methodological introduction of the consensus

13. Hämmerle CH, Klinge B, Quirynen M. The 4th EAO Consensus Conference 11-14 February 2015, Pfäffikon, Schwyz, Switzerland. Clin Oral Implants Res. 2015;26(11):3-4.

Reason: Introductory part of the consensus

14. Beikler T, Flemmig TF. EAO consensus conference: economic evaluation of implant-supported prostheses. Clin Oral Implants Res. 2015;26(11):57-63.

Reason: Narrative review to explain the consensus

15. Terheyden H, Kopp I. Proceedings of the 1st Consensus Conference of the German Association of Oral Implantology, Aerzen, Germany, 29th and 30th September 2010 Introduction, background and methodology. Eur J Oral Implantol.

2011;4:5-10.

Reason: Introductory part of the consensus

16. Pini-Prato G, Nieri M, Pagliaro U, Giorgi TS, La Marca M, Franceschi D, Buti J, Giani M, Weiss JH, Padeletti L, Cortellini P, Chambrone L, Barzagli L, Defraia E, Rotundo R; National Association of Italian Dentists (ANDI)--Tuscany Region. Surgical treatment of single gingival recessions: clinical guidelines. Eur J Oral Implantol. 2014;7(1):9-43.

Reason: Document not related to implant dentistry

17. Klineberg I, Cameron A, Hobkirk J, Bergendal B, Maniere MC, King N, Watkins S, Hobson R, Stanford C, Kurtz K, Sharma A. Rehabilitation of children with ectodermal dysplasia. Part 2: an international consensus meeting. Int J Oral Maxillofac Implants. 2013;28(4):1101-9.

Reason: Document not related to implant dentistry

18. Klineberg I, Cameron A, Whittle T, Hobkirk J, Bergendal B, Maniere MC, King N, Palmer R, Hobson R, Stanford C, Kurtz K, Sharma A, Guckes A. Rehabilitation of children with ectodermal dysplasia. Part 1: an international Delphi study. Int J Oral Maxillofac Implants. 2013;28(4):1090-1100.

Reason: Document not related to implant dentistry
