# Supplementary material for: Methodological Quality of Consensus Guidelines in Implant Dentistry
Source: PLoS One. 2017 Jan 20;12(1):e0170262. doi: 10.1371/journal.pone.0170262 (PMC5249121; doi:10.1371/journal.pone.0170262)
Supplement: S1 Table — (DOCX) [file pone.0170262.s004.docx]

**S1 Table**. Evaluation of consensus guidelines published in high-ranked implant dentistry journals with the AGREE II instrument

| DOCUMENT | DOMAIN 1 | | | DOMAIN 2 | | | DOMAIN 3 | | | | | | | | DOMAIN 4 | | | DOMAIN 5 | | | | DOMAIN 6 | |
| --- | --- | --- | --- | --- | --- | --- | --- | --- | --- | --- | --- | --- | --- | --- | --- | --- | --- | --- | --- | --- | --- | --- | --- |
|  | AGREE 1 | AGREE 2 | AGREE 3 | AGREE 4 | AGREE 5 | AGREE 6 | AGREE 7 | AGREE 8 | AGREE 9 | AGREE 10 | AGREE 11 | AGREE 12 | AGREE 13 | AGREE 14 | AGREE 15 | AGREE 16 | AGREE 17 | AGREE 18 | AGREE 19 | AGREE 20 | AGREE 21 | AGREE 22 | AGREE 23 |
| CAMLOG 2014 | 14 | 15 | 15 | 16 | 16 | 4 | 16 | 10 | 14 | 11 | 11 | 20 | 11 | 17 | 18 | 9 | 21 | 8 | 15 | 11 | 7 | 10 | 11 |
| CAMLOG 2016 | 17 | 12 | 12 | 18 | 8 | 16 | 15 | 12 | 8 | 8 | 17 | 21 | 12 | 15 | 19 | 11 | 22 | 9 | 15 | 10 | 7 | 11 | 12 |
| CIDRR 2012 | 11 | 9 | 14 | 20 | 17 | 19 | 8 | 7 | 12 | 19 | 24 | 13 | 4 | 4 | 24 | 21 | 23 | 5 | 21 | 17 | 4 | 4 | 5 |
| EAO 2015 WG 1 | 28 | 26 | 16 | 20 | 5 | 12 | 24 | 19 | 24 | 26 | 28 | 23 | 10 | 4 | 23 | 16 | 27 | 4 | 4 | 19 | 4 | 23 | 24 |
| EAO 2015 WG 2 | 25 | 21 | 24 | 20 | 4 | 12 | 4 | 4 | 9 | 26 | 26 | 27 | 16 | 4 | 22 | 23 | 27 | 16 | 23 | 8 | 4 | 24 | 4 |
| EAO 2015 WG 3 | 8 | 20 | 20 | 25 | 4 | 12 | 28 | 12 | 24 | 24 | 25 | 28 | 17 | 4 | 22 | 24 | 26 | 20 | 24 | 8 | 4 | 24 | 4 |
| EAO 2015 WG 4 | 9 | 17 | 12 | 20 | 12 | 12 | 27 | 25 | 24 | 24 | 26 | 28 | 16 | 4 | 23 | 24 | 27 | 17 | 22 | 7 | 4 | 24 | 4 |
| EAO 2012 WG 1 | 25 | 24 | 17 | 15 | 13 | 4 | 6 | 20 | 8 | 4 | 21 | 35 | 16 | 10 | 24 | 21 | 25 | 13 | 4 | 10 | 12 | 4 | 4 |
| EAO 2012 WG 2 | 24 | 22 | 16 | 14 | 14 | 4 | 8 | 5 | 9 | 4 | 11 | 19 | 18 | 10 | 22 | 11 | 23 | 12 | 4 | 8 | 9 | 17 | 4 |
| EAO 2012 WG 3 | 24 | 24 | 14 | 14 | 12 | 4 | 7 | 9 | 13 | 5 | 10 | 17 | 16 | 8 | 25 | 18 | 24 | 13 | 4 | 8 | 19 | 4 | 6 |
| EAO 2012 WG4 | 24 | 23 | 17 | 15 | 9 | 4 | 11 | 13 | 18 | 9 | 8 | 16 | 16 | 7 | 24 | 22 | 24 | 13 | 4 | 8 | 16 | 4 | 6 |
| EAO 2011 | 17 | 21 | 20 | 23 | 17 | 26 | 4 | 4 | 6 | 10 | 26 | 18 | 8 | 8 | 22 | 21 | 23 | 19 | 24 | 11 | 4 | 18 | 23 |
| EJOI 2012 | 27 | 24 | 24 | 24 | 24 | 24 | 26 | 24 | 25 | 26 | 26 | 25 | 5 | 4 | 25 | 24 | 26 | 12 | 17 | 5 | 4 | 19 | 22 |
| EJOI 2014 | 4 | 4 | 9 | 15 | 15 | 12 | 4 | 4 | 9 | 4 | 17 | 22 | 4 | 5 | 9 | 13 | 15 | 4 | 5 | 4 | 5 | 4 | 5 |
| ESTEPONA 2012 | 7 | 6 | 7 | 14 | 5 | 5 | 4 | 5 | 6 | 4 | 5 | 14 | 5 | 4 | 11 | 13 | 16 | 5 | 18 | 5 | 4 | 6 | 5 |
| IMPLANT DENTISTRY 2012 | 18 | 14 | 14 | 20 | 14 | 20 | 18 | 6 | 9 | 16 | 24 | 13 | 12 | 8 | 24 | 20 | 23 | 10 | 20 | 17 | 5 | 17 | 17 |
| ITI 2014 WG1 | 20 | 23 | 22 | 18 | 9 | 21 | 5 | 4 | 4 | 24 | 8 | 13 | 5 | 4 | 27 | 15 | 21 | 5 | 11 | 4 | 26 | 27 | 27 |
| ITI 2014 WG2 | 20 | 24 | 22 | 18 | 8 | 22 | 5 | 4 | 4 | 24 | 6 | 19 | 4 | 4 | 28 | 15 | 22 | 4 | 8 | 4 | 24 | 27 | 27 |
| ITI 2014 WG3 | 22 | 21 | 21 | 18 | 9 | 19 | 5 | 4 | 4 | 21 | 8 | 16 | 5 | 4 | 28 | 16 | 22 | 4 | 8 | 4 | 24 | 27 | 27 |
| ITI 2014 WG4 | 24 | 24 | 24 | 14 | 9 | 21 | 7 | 5 | 4 | 21 | 7 | 15 | 4 | 4 | 27 | 15 | 21 | 5 | 8 | 4 | 24 | 27 | 28 |
| ITI 2014 WG5 | 25 | 23 | 24 | 14 | 9 | 20 | 5 | 4 | 4 | 24 | 8 | 16 | 5 | 4 | 28 | 15 | 21 | 4 | 10 | 4 | 24 | 28 | 27 |
| OSTEOLOGY 2012 | 28 | 24 | 24 | 24 | 5 | 13 | 22 | 21 | 22 | 22 | 23 | 24 | 23 | 12 | 23 | 24 | 24 | 16 | 20 | 12 | 5 | 24 | 27 |
| GUIDELINES AO 2010 | 27 | 10 | 14 | 16 | 7 | 14 | 7 | 4 | 6 | 4 | 6 | 11 | 6 | 15 | 22 | 16 | 21 | 6 | 18 | 15 | 14 | 18 | 13 |
