# Supplementary material for: Methodological Quality of Consensus Guidelines in Implant Dentistry
Source: PLoS One. 2017 Jan 20;12(1):e0170262. doi: 10.1371/journal.pone.0170262 (PMC5249121; doi:10.1371/journal.pone.0170262)
Supplement: S2 Table — (DOCX) [file pone.0170262.s005.docx]

**S2 Table**. Evaluation of consensus guidelines + systematic review published in high-ranked implant dentistry journals with the AGREE II instrument

|  | DOMAIN 1 | | | DOMAIN 2 | | | DOMAIN 3 | | | | | | | | DOMAIN 4 | | | DOMAIN 5 | | | | DOMAIN 6 | |
| --- | --- | --- | --- | --- | --- | --- | --- | --- | --- | --- | --- | --- | --- | --- | --- | --- | --- | --- | --- | --- | --- | --- | --- |
| Document | AGREE 1 | AGREE 2 | AGREE 3 | AGREE 4 | AGREE 5 | AGREE 6 | AGREE 7 | AGREE 8 | AGREE 9 | AGREE 10 | AGREE 11 | AGREE 12 | AGREE 13 | AGREE 14 | AGREE 15 | AGREE 16 | AGREE 17 | AGREE 18 | AGREE 19 | AGREE 20 | AGREE 21 | AGREE 22 | AGREE 23 |
| CAMLOG 2014 | 15 | 18 | 19 | 17 | 21 | 9 | 21 | 18 | 17 | 16 | 19 | 24 | 17 | 17 | 21 | 17 | 23 | 11 | 23 | 11 | 7 | 19 | 21 |
| CAMLOG 2016 | 19 | 20 | 18 | 18 | 11 | 19 | 19 | 20 | 15 | 17 | 19 | 23 | 17 | 13 | 20 | 17 | 23 | 10 | 22 | 10 | 7 | 19 | 21 |
| JOMI 2012 |  |  |  |  |  |  |  |  |  |  |  |  |  |  |  |  |  |  |  |  |  |  |  |
| EAO 2015 WG 1 | 28 | 28 | 18 | 24 | 28 | 21 | 27 | 28 | 24 | 28 | 28 | 28 | 16 | 20 | 24 | 16 | 28 | 11 | 19 | 20 | 21 | 28 | 28 |
| EAO 2015 WG 2 | 26 | 24 | 26 | 24 | 5 | 19 | 27 | 26 | 25 | 28 | 28 | 27 | 24 | 7 | 24 | 25 | 28 | 20 | 27 | 24 | 4 | 24 | 6 |
| EAO 2015 WG 3 | 27 | 23 | 24 | 26 | 24 | 24 | 28 | 28 | 28 | 24 | 25 | 28 | 23 | 4 | 24 | 24 | 28 | 24 | 24 | 23 | 5 | 26 | 24 |
| EAO 2015 WG 4 | 27 | 24 | 20 | 21 | 21 | 21 | 28 | 28 | 28 | 27 | 28 | 28 | 23 | 6 | 28 | 28 | 28 | 23 | 24 | 23 | 6 | 25 | 24 |
| EAO 2012 WG 1 | 25 | 26 | 20 | 14 | 13 | 4 | 17 | 21 | 11 | 5 | 21 | 20 | 16 | 10 | 25 | 23 | 27 | 14 | 4 | 11 | 12 | 5 | 12 |
| EAO 2012 WG 2 | 27 | 26 | 17 | 18 | 15 | 7 | 18 | 12 | 9 | 4 | 11 | 20 | 18 | 10 | 25 | 13 | 23 | 13 | 4 | 7 | 8 | 13 | 4 |
| EAO 2012 WG 3 | 26 | 26 | 13 | 16 | 14 | 4 | 17 | 13 | 13 | 9 | 9 | 19 | 16 | 10 | 25 | 23 | 24 | 12 | 4 | 8 | 16 | 5 | 8 |
| EAO 2012 WG4 | 26 | 27 | 19 | 19 | 14 | 4 | 19 | 16 | 8 | 9 | 9 | 19 | 16 | 8 | 22 | 23 | 24 | 12 | 4 | 8 | 16 | 5 | 8 |
| EAO 2012 |  |  |  |  |  |  |  |  |  |  |  |  |  |  |  |  |  |  |  |  |  |  |  |
| EJOI 2011 WG1 | 28 | 28 | 28 | 28 | 28 | 23 | 28 | 24 | 18 | 28 | 28 | 28 | 16 | 20 | 19 | 13 | 28 | 5 | 4 | 5 | 4 | 24 | 24 |
| EJOI 2011 WG2 | 27 | 28 | 27 | 27 | 27 | 24 | 27 | 24 | 20 | 22 | 26 | 27 | 16 | 23 | 24 | 17 | 27 | 4 | 6 | 6 | 5 | 24 | 22 |
| EJOI 2011 WG3 | 27 | 27 | 27 | 27 | 26 | 16 | 27 | 25 | 26 | 27 | 23 | 28 | 22 | 20 | 23 | 15 | 27 | 6 | 4 | 4 | 5 | 22 | 23 |
| EJOI 2011 WG4 | 28 | 28 | 25 | 27 | 28 | 24 | 7 | 6 | 5 | 27 | 26 | 27 | 18 | 24 | 21 | 13 | 28 | 4 | 4 | 5 | 4 | 24 | 23 |
| EJOI 2012 | 27 | 25 | 26 | 26 | 27 | 25 | 27 | 28 | 27 | 28 | 27 | 27 | 10 | 4 | 28 | 26 | 28 | 12 | 18 | 18 | 4 | 21 | 24 |
| EJOI 2014 | 25 | 25 | 25 | 19 | 17 | 17 | 21 | 21 | 21 | 8 | 20 | 24 | 10 | 4 | 11 | 17 | 18 | 5 | 6 | 6 | 5 | 8 | 10 |
| ESTEPONA 2012 | 11 | 9 | 13 | 19 | 13 | 16 | 9 | 6 | 12 | 17 | 18 | 15 | 6 | 6 | 23 | 21 | 23 | 6 | 21 | 12 | 5 | 5 | 5 |
| IMPLANT DENTISTRY 2012 |  |  |  |  |  |  |  |  |  |  |  |  |  |  |  |  |  |  |  |  |  |  |  |
| ITI 2014 WG1 | 25 | 24 | 24 | 16 | 7 | 24 | 24 | 21 | 24 | 23 | 7 | 15 | 5 | 4 | 27 | 19 | 21 | 8 | 11 | 5 | 22 | 26 | 28 |
| ITI 2014 WG2 | 25 | 24 | 23 | 16 | 6 | 23 | 24 | 23 | 23 | 23 | 7 | 15 | 4 | 4 | 27 | 17 | 22 | 8 | 9 | 4 | 20 | 25 | 28 |
| ITI 2014 WG3 | 25 | 24 | 24 | 13 | 4 | 24 | 25 | 21 | 22 | 23 | 7 | 17 | 4 | 5 | 28 | 17 | 20 | 8 | 8 | 4 | 21 | 24 | 28 |
