# Supplementary material for: Methodological Quality of Consensus Guidelines in Implant Dentistry
Source: PLoS One. 2017 Jan 20;12(1):e0170262. doi: 10.1371/journal.pone.0170262 (PMC5249121; doi:10.1371/journal.pone.0170262)
Supplement: S3 Table — IQR: interquartile range. CG: consensus guideline; CGSR: consensus guideline + systematic review. (DOCX) [file pone.0170262.s006.docx]

**S3 Table**. Medians of percentages of the maximum possible score for the respective domains across consensus guidelines in implant dentistry (19 possible comparisons). IQR: interquartile range. CG: consensus guideline; CGSR: consensus guideline + systematic review.

| **Domains** | Median of % (IQR) | Median of % (IQR) |
| --- | --- | --- |
|  | CG | CGSR |
| 1. Scope and purpose | 72.22 (36.11) | 83.33 (5.56) |
| 2. Stakeholder involvement | 41.67 (16.67) | 48.61 (16.67) |
| 3. Rigour of development | 34.38 (46.35) | 50 (33.33) |
| 4. Clarity of presentation | 75 (20.83) | 77.78 (15.28) |
| 5. Applicability | 26.04 (8.33) | 29.17 (18.75) |
| 6. Editorial independence | 41.67 (89.58) | 77.08 (77.08) |
