# Supplementary material for: Methodological Quality of Consensus Guidelines in Implant Dentistry
Source: PLoS One. 2017 Jan 20;12(1):e0170262. doi: 10.1371/journal.pone.0170262 (PMC5249121; doi:10.1371/journal.pone.0170262)
Supplement: S4 Table — (DOCX) [file pone.0170262.s007.docx]

**S4 Table**. Percentage of the maximum possible score for the respective domains across consensus guidelines in implant dentistry

| **Consensus guidelines** | **Domain 1** | | **Domain 2** | | **Domain 3** | | **Domain 4** | | **Domain 5** | | **Domain 6** | |
| --- | --- | --- | --- | --- | --- | --- | --- | --- | --- | --- | --- | --- |
|  | CG | CGSR | CG | CGSR | CG | CGSR | CG | CGSR | CG | CGSR | CG | CGSR |
| Schwarz et al 2016 | 40.3 | 62.5 | 41.7 | 50.0 | 39.6 | 57.8 | 55.6 | 66.7 | 26.0 | 34.4 | 31.3 | 66.7 |
| Klinge et al 2015 | 80.6 | 86.1 | 34.7 | 84.7 | 65.6 | 87.0 | 75.0 | 77.8 | 15.6 | 57.3 | 81.3 | 100.0 |
| Hammerle et al 2015 | 80.6 | 88.9 | 33.3 | 50.0 | 43.8 | 83.3 | 83.3 | 90.3 | 36.5 | 61.5 | 41.7 | 45.8 |
| Sicilia et al 2015 | 500 | 86.1 | 40.3 | 86.1 | 67.7 | 81.3 | 83.3 | 88.9 | 41.7 | 62.5 | 41.7 | 87.5 |
| Sanz et al 2015 | 36.1 | 81.9 | 44.4 | 70.8 | 74.0 | 85.4 | 86.1 | 100.0 | 35.4 | 62.5 | 41.7 | 85,4 |
| Schwarz et al 2014 | 44.4 | 55.6 | 33.3 | 48.6 | 40.6 | 60.9 | 50.0 | 68.1 | 26.0 | 37.5 | 27.1 | 66.7 |
| FOR 2014 | 6.9 | 87.5 | 41.7 | 56.9 | 19.3 | 50.5 | 34.7 | 47.2 | 2.1 | 6.3 | 2.1 | 20.8 |
| Bornstein et al 2014 | 73.6 | 84.7 | 50.0 | 48.6 | 18.2 | 47.4 | 70.8 | 76.4 | 31.3 | 31.3 | 95.8 | 95.8 |
| Wismeijer et al 2014 | 75.0 | 83.3 | 50.0 | 45.8 | 19.8 | 47.4 | 73.6 | 75.0 | 25.0 | 26.0 | 95.8 | 93.8 |
| Morton et al 2014 | 72.2 | 84.7 | 47.2 | 40.3 | 18.2 | 47.9 | 75.0 | 73.6 | 25.0 | 26.0 | 95.8 | 91.7 |
| Gallucci et al 2014 | 83.3 | 81.9 | 44.4 | 41.7 | 18.2 | 46.9 | 70.8 | 77.8 | 26.0 | 25.0 | 97.9 | 97.9 |
| Heitz-mayfield et al 2014 | 83.3 | 83.3 | 43.1 | 40.3 | 19.8 | 50.0 | 72.2 | 75.0 | 27.1 | 27.1 | 97.9 | 97.9 |
| Albrektsson et al 2012 | 11,1 | 29.2 | 16.7 | 50.0 | 7.8 | 29.7 | 38.9 | 76.4 | 16.7 | 29.2 | 6.3 | 4.2 |
| Harris et al 2011 | 63.9 |  | 75.0 |  | 27.1 |  | 75.0 |  | 43.8 |  | 68.8 |  |
| Albrektsson et al 2012 | 75.0 | 81.9 | 27.8 | 26.4 | 45.8 | 46.4 | 80.6 | 87.5 | 24.0 | 26.0 | 0.0 | 18.8 |
| Klinge et al 2012 | 69.4 | 80.6 | 27.8 | 38.9 | 27.1 | 36.5 | 61.1 | 68.1 | 17.7 | 16.7 | 27.1 | 18.8 |
| Sicilia et al 2012 | 69.4 | 73.6 | 25.0 | 30.6 | 27.6 | 38.5 | 76.4 | 83.3 | 29.2 | 25.0 | 4.2 | 10.4 |
| Gotfredsen et al 2012 | 72.2 | 83.3 | 22.2 | 34.7 | 34.4 | 37.5 | 80.6 | 79.2 | 26.0 | 25.0 | 4.2 | 10.4 |
| Benavides et al 2012 | 47.2 |  | 58.3 |  | 38.5 |  | 76.4 |  | 37.5 |  | 54.2 |  |
| Hammerle et al 2012 | 88.9 | 91.7 | 41.7 | 47.2 | 71.4 | 79.7 | 81.9 | 90.3 | 38.5 | 43.8 | 89.6 | 100.0 |
| Esposito et al 2012 | 87.5 | 91.7 | 83.3 | 91.7 | 67.2 | 76.0 | 87.5 | 97.2 | 22.9 | 37.5 | 68.8 | 77.1 |
| Academy of Osseointegration 2010 | 54.2 |  | 34.7 |  | 14.1 |  | 65.3 |  | 38.5 |  | 47.9 |  |
| Klein et al 2011 |  | 100.0 |  | 93.1 |  | 82.3 |  | 66.7 |  | 2.1 |  | 83.3 |
| Schley et al 2011 |  | 97.2 |  | 91.7 |  | 79.7 |  | 77.8 |  | 5.2 |  | 79.2 |
| Nitsche et al 2011 |  | 95.8 |  | 79.2 |  | 86.5 |  | 73.6 |  | 3.1 |  | 77.1 |
| Weng et al 2011 |  | 95.8 |  | 93.1 |  | 56.3 |  | 69.4 |  | 1.0 |  | 81.3 |
| Albrektsson et al 2012 | 30.6 |  | 61.1 |  | 30.7 |  | 77.8 |  | 32.3 |  | 2.1 |  |

CG: consensus guideline

CGSR: consensus guideline + systematic review
